# Differential CD30 expression in adult T-cell leukemia-lymphoma subtypes

**DOI:** 10.1186/1742-4690-11-S1-P129

**Published:** 2014-01-07

**Authors:** Germán Campuzano-Zuluaga, Agustin Pimentel, Jennifer R Chapman-Fredricks, Juan Carlos Ramos

**Affiliations:** 1 Department of Pathology and Laboratory Medicine, University of Miami Miller School of Medicine and Jackson Memorial Hospital, Miami, FL, USA; 2 Division of Hematology-Oncology, Department of Medicine, Sylvester Comprehensive, University of Miami, Miami, FL, USA

## 

Adult T-cell leukemia-lymphoma (ATLL) is caused by HTLV-I, and is a disease with dismal prognosis urging new therapies. Brentuximab vedotin (SGN-35) is an anti-CD30 monoclonal antibody conjugated to a potent microtubule poisoning agent monomethyl auristatin E that is effective in the treatment of CD30-expressing lymphomas. There are conflicting reports on the frequency of ATLL tumors expressing CD30. At our institution we encounter a relatively large number of ATLL cases due to our patient demographics. Thirty-nine out of 156 ATLL cases identified so far have been checked for CD30 status. CD30 expression was evaluated by immunohistochemistry (IHC) performed on either tissue sections as part of the routine pathology workup or cytospins prepared from CD4+ lymphocyte-enriched peripheral blood specimens using 20% expression as a cut-off positive value. The proportion of CD30+ ATLLs was 36% (95% CI 11%-61%), including 47% in lymphomatous-type, 28% in acute-type, and 10% in indeterminate cases. Four of 12 (33%) acute-type ATLL cytospin cases were CD30+, however, a high expression of 80% was observed in only one case. One patient with CD30+ acute-type ATLL with diffuse skin involvement treated with brentuximab had an objective transient response. Our preliminary data show that 36% of ATLLs are CD30+ and therefore may be amenable to anti-CD30 therapy. However, the low percentage of CD30+ cells in acute-type ATLL may limit the response to anti-CD30 target therapies for this subtype. We are currently testing a larger number of tissue and leukemic ATLL specimens available for further immunophenotypic characterization and comprehensive analysis of clinical outcome.

